# Diffusion of Carbon Monoxide and Hydrogen Cyanide to Muscles and Blood—An Experimental Study

**DOI:** 10.3390/toxics10110707

**Published:** 2022-11-18

**Authors:** Jacek Baj, Grzegorz Buszewicz, Dominika Przygodzka, Alicja Forma, Jolanta Flieger, Grzegorz Teresiński

**Affiliations:** 1 Department of Human Anatomy, Medical University of Lublin, Jaczewskiego 4, 20-090 Lublin, Poland; 2 Department of Forensic Medicine, Medical University of Lublin, Jaczewskiego 8b, 20-090 Lublin, Poland; 3 Department of Analytical Chemistry, Medical University of Lublin, Chodźki 4A, 20-093 Lublin, Poland

**Keywords:** combustion gas poisoning, hydrogen cyanide, carboxyhemoglobin, carboxymyoglobin, post-mortem diffusion

## Abstract

Postmortem carbon monoxide (CO) and hydrogen cyanide (HCN) diffusion under ambient conditions was assessed in a human cadaver model. The main objective of this study was to determine whether the postmortem diffusion of HCN and CO greatly affected the determination of HCN, carboxyhemoglobin (COHb), and carboxymyoglobin (COMb). Layered samples of blood, musculocutaneous, and muscular specimens were collected from the adult cadavers and placed in the tight chambers designed for the purpose of this experiment. The specimens were treated with CO and HCN for 24 h. COHb and COMb were determined using headspace gas chromatography (GC) with an O-FID detector while the HCN values were assessed using a GC headspace with an NPD detector. It was shown that the skin substantially limited the diffusion of CO which penetrated the superficial layers of the muscle very slightly, all the while not affecting the blood level of COHb in the 4.5 cm layer of the muscle located underneath. There were no differences regarding the CO diffusion between superficially charred and thermally coagulated compared to that observed in intact integuments. In addition, the cutaneous sample deprived of the adipose layer was not shown to be a barrier to the moderate diffusion of CO into the blood layer below. HCN was found to easily diffuse from the skin to the blood vessels (vein specimens), and partial charring and thermocoagulation of the superficial muscular layer favored the diffusion of cyanides into the tissues. Similarly to CO, HCN diffusion to the blood and muscles was greatly limited by the adipose layer.

## 1. Introduction

Due to the combustion of organic substances, wool, plastics, etc., toxic gases, such as carbon monoxide (CO) and hydrogen cyanide (HCN) are produced [1]. Many fire-related fatalities are caused by one or both of these gases. The toxic effects of HCN and CO are additive; however, both contribute to tissue hypoxia via different mechanisms [2,3,4,5]. In cases of charred corpses that are found at the site of a fire, forensic experts must determine whether the victim was alive when the fire started, since there are cases wherein a fire was started in order to cover up criminal traces, such as homicide, for example. Diagnostic procedures for HCN poisoning (for forensic purposes) involve determinations of cyanide (total or free form) in blood and tissues by various methods [6]. CO poisonings are diagnosed by determining the percentage of COHb [7,8,9,10]; whereas, in the case of partial charring of corpses and thermal coagulation of blood, CO poisonings can be diagnosed by carboxymyoglobin determinations in specimens of the deeper layers of muscles [11]. However, CO affinity for myoglobin is a few times lower than for hemoglobin. Both CO and HCN are lighter than air. HCN is soluble in water (in the form of weak hydrocyanic acid) while CO is slightly soluble.

### 1.1. CO Diffusion

Examples of easy CO penetration through gypsum wallboards have been reported [12]. The penetration ability of atmospheric CO into the tissues is commonly used in the food industry to stabilize the color of the meat [13]. In living subjects, CO absorption in blood through the pulmonary alveoli is well studied and known to be extremely rapid, while its possible penetration through the skin integuments is largely overlooked. However, in many older German sources, authors have recommended sampling blood for COHb analysis from corpses exposed to CO atmospheres from cardiac cavities and not from peripheral veins, due to the risk of diagnostic errors related to passive diffusion through the integuments [14,15,16]. We are aware of only one such recommendation in the older English textbooks [17]. The current scientific literature generally does not mention recommending sampling from cardiac cavities; however, this practice is still used in many forensic institutions nowadays.

Historical background: The issue of postmortem CO diffusion through integuments and into soft tissues was first highlighted by Wachholz and Lemberger [18,19], who placed the corpses of newborns in containers filled with pure CO and observed significant changes in livor color after only a 30 min incubation. Their findings were corroborated by Mirto, Dominicis, Strassmann, and Stoll [19,20,21]; however, they also used the corpses of newborns and infants and determined COHb qualitatively only. Schwarzacher and Reuter [22,23] have demonstrated that after a 14 h exposure of corpses to CO, the gas permeates the superficial veins and superficial muscular layers exclusively. Moreover, Breitenecker has found that after a 48 h CO incubation period, a 2–5% concentration of COHb was found in the heart and cranial sinuses, and a 75% concentration was found in superficial subcutaneous veins [14].

### 1.2. HCN Diffusion

Cyanide may be one of the major contributors to mortality observed in approximately 3000–6000 deaths from home structure fires occurring in the years 1980–2021 in the United States [24]. Many synthesized (melamine, polyamide, polyurethane, polyacrylonitrile, urea–formaldehyde) and natural (e.g., wool, silk) compounds may release HCN during burning [25]. Importantly, HCN can also be absorbed through the skin [26,27]. Drinker [28] and Potter [29] have reported that workers exposed to HCN became dizzy and fell unconscious despite wearing gas masks providing respiratory protection. The pulmonary absorption of HCN is much faster compared to the dermal absorption; the amount and speed of absorption through the human skin depend on the amount of skin moisture as well as the duration of skin contact [27,30]. An average LD 50 value for dermal exposure of 100 mg/kg body weight was estimated for humans [31]. Concentrations of 7000–12,000 mg/m^3^ were shown to be fatal after a 5 min exposure of workers with self-contained respirators that were not provided with an effective skin protection [32]. Moreover, cases of postmortem CO diffusion have also been reported [33]. On the other hand, no HCN nor carboxymyoglobin have been found in the blood samples collected from corpses burned after death (homicide, suicide, accident) [34].

The purpose of the study was to determine whether the postmortem diffusion of gases affected HCN, COMb, and COHb levels in blood and tissues of corpses exposed to HCN and CO in the atmosphere. The study design was approved by the local bioethics committee (KE-0254/217/2017).

## 2. Material and Methods

### 2.1. The Study Material

Blood from the femoral vein, fragments of skin from the lumbar region with the adipose tissue, sections from the femoral muscle, and fragments of the great saphenous vein showing no putrefaction changes were collected from adults about 24–48 h after death. Fragments of the vessels of an approximate length of 2 cm were filled with blood (about 1 mL) and tied at both ends. Toxicology tests were performed (ordered by the prosecutors’ offices) and no concentrations of COHb and COMb as well as HCN were detected, which excluded CO and HCN poisoning. The tissues were cut into cuboids adjusted to the experimental chamber. The superficially charred muscle was obtained from the muscle specimen charred on an electric heat plate until the thickness of macroscopically visible coagulation was 0.5 cm and cut into 4 × 4 × 4.5 cm pieces.

### 2.2. Chemicals

Formic acid 80% (Merck, Darmstadt, Germany), H_2_SO_4_ 96% pure for analysis (POCH, Gliwice, Poland), potassium cyanide (Aldrich, St. Louis, MO, USA), polysiloxane-stable pasta, and neutral colorless (Soudal Pty Ltd., Glendenning, Australia) were used.

### 2.3. Analytical Equipment

Gas chromatograph Trace GC-Ultra with headspace autosampler Tri-Plus (Thermo-Finnigan, San Jose, CA, USA) was used. Capillary columns and detectors used were as follows: COHb/COMb determination—Molesiv 0.5 mm × 30 m (Agilent J&W, Santa Clara, CA, USA), O-FID detector (methanizer-equipped FID), HCN determination—GS-Q (Agilent J&W, Santa Clara, CA, USA), and NPD detector.

### 2.4. Experimental Equipment

**Figure 1:**
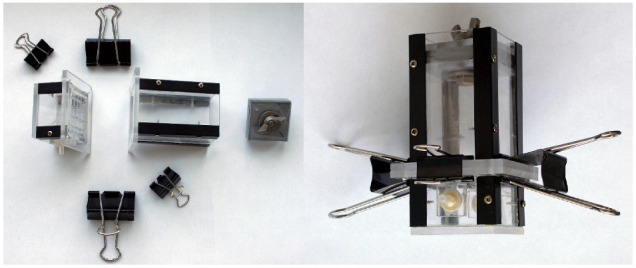
The chamber for CO experiments before and after assembly.

Chambers used for exposure of the study material to CO and HCN effects were constructed from 4 mm thick plexiglass slabs (Figure 1).

**Figure 2:**
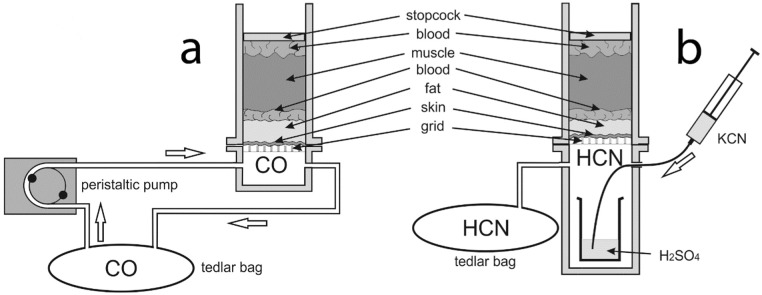
A diagram of CO (**a**) and HCN (**b**) experiments.

The 4 × 4 cm chambers were composed of 2 parts: an upper and a lower part. The study material was placed in the upper part, 10 cm in height. The specimen borders were sealed along the chamber walls (from the side of the gas-filled chamber) using polysiloxane-stable pasta. The chamber was closed superiorly with a tight stopcock. The lower chamber was filled with either CO or HCN. Both parts were separated by a grid in order to securely contain the material. The lower chamber for the CO experiments was 2 cm high and had inlets and outlets through which CO was pumped using a peristaltic pump (Figure 2a). A 1.5 L Tedlar sampling bag (Merck, Darmstadt, Germany) was used to capture and use >90% CO formed via the dehydration of 80% formic acid (25 mL) in the presence of 96% sulfuric acid (12.5 mL, added dropwise) [35]. Before the experiment, the air from the Tedlar bag was removed using a vacuum pump—the effect of air deposition was therefore scarce (negligible). Throughout the experiment, gas circulated in a closed loop between the chamber and the bag with a flow rate of 6 mL/min. The lower chamber for the HCN experiments was 10 cm high in order to hold a 10 mL glass beaker with 5 mL of concentrated H_2_SO_4_. During the experiment, the chamber was placed on a thermoblock maintaining the temperature of +30 °C in order to increase the efficiency of the reaction. A 1 mL of 3% KCN solution (in water) was injected into the beaker with sulfuric acid through the inlet, which decomposed into HCN and K_2_SO_4_. Excessive HCN was poured into the Tedlar bag through the outlet (Figure 2).

## 3. The Course of the Experiment

The tissue specimens were placed in the experimental chamber and sealed with polysiloxane pasta. 

The following experimental patterns were used:

1. A 4 × 4 cm skin fragment of fatty tissue (approx. 1.5 cm in depth)—a fragment of vessel with blood–muscle (approx. 4.5 cm in depth)—a second fragment of the vessel with blood);
2. Skin (without subcutaneous tissue)—vessel with blood–muscle–vessel;
3. Muscle–vessel with blood;
4. Superficially charred muscle–vessel with blood.

The specimens were exposed to CO at +25 °C and to HCN at +30 °C (above the boiling point of HCN, +26 °C). Four runs were made for each experimental system. The exposure was discontinued after 24 h, the veins filled with blood were removed and the muscle was immediately frozen in the chamber at −20 °C. The 5 mm peripheral layers were discarded. From the remaining muscular part, three 1 cm segments were cut, starting from the lower side more exposed to the gas (symbolic layers 1, 2, and 3). Concentrations of COMb or HCN (depending on the gas used) were determined 3 times in each sample to quantify the depth of possible diffusion. COHB and COMb were determined using a GC headspace with an O-FID detector [9,11], whereas levels of HCN in blood and muscles were determined using a GC headspace with an NPD detector [36]. Both methods were validated for routine (forensic) analysis.

## 4. Results

Table 1 presents the results of COHb and COMb determinations (% sat.) and Table 2 presents levels of HCN (blood: mg/L, muscle: mg/kg).

During the experiment with CO, a gradual change in the colors of the muscle into bright red from the side flushed with the gas was visible (Figure 3 and a Video S1 available online).

In tests 2–4, the coloration reached the maximum depth of about 1–1.5 cm. Concerning the samples without a subcutaneous fatty tissue layer, the mean blood concentration of COHb in the vessels located under the skin was 19% (range 7–28%) and of HCN was 38.97 mg/L (20.50–48.91 mg/L). In the superficial muscle layer, the mean HCN concentration was 2.96 mg/kg, at a depth of approximately 2 cm it was 0.94 mg/L, and at a depth of approximately 3 cm it was very low—below the limit of quantitation (LOQ = 0.02 mg/kg) in three out of four samples.

## 5. Discussion

The major objective of this experiment was answering the question of whether the postmortem diffusion of CO/HCN could affect the determination of COHB/COMB and HCN in the corpses that were found at the site of fire after death. In case of a living (breathable) victim, diffusion through the skin layers had a very slight influence compared to the pulmonary absorption [37].

The results of our experiments demonstrate that even a modest adipose tissue thickness of approximately 1.5 cm collected from normosthenic corpses excellently prevents CO diffusion. The 24 h exposure to >90% CO resulted only in trace CO hemoglobin saturation. Likewise, trace saturation of myoglobin was also observed in the superficial muscle layer. The isolating properties of the skin denudated of subcutaneous tissue by scraping were markedly worse: in blood, about 7–28% COHb, while in the superficial muscle layer, about 1–3% COMb. A relatively wide range of COHb saturation in the examined samples may be related to individual properties of donor subjects, e.g., variability of skin thickness. When the unprotected muscle was exposed (Table 1, patterns 3), CO diffusion was limited to the superficial layer, demonstrating that the 1 cm layer of muscle effectively protects against CO diffusion deep into the tissues.

The hemoglobin saturation with CO was found to be greatly higher compared to the myoglobin saturation. The above can be associated with the higher CO binding affinity of hemoglobin, which contains four particles of heme (myoglobin only contains one). Charring and thermal coagulation of the superficial muscle layer only slightly increased myoglobin saturation with CO in the muscle layers below the coagulated region. It should be strongly emphasized that in the blood located under the 4.5 cm muscle layer, even when superficially coagulated (experiments 4), the concentrations of COHb did not increase to the levels that could be considered toxic in real cases.

It was shown that HCN is quite easily diffused through the superficial skin into subcutaneous veins, and, notably, readily permeated into the deeper layers of denuded muscles. HCN, in the presence of water, forms weak hydrocyanic acid (prussic acid), which diffuses through the tissues; hence the high concentrations of cyanides in highly hydrated blood (about 80% H_2_0) and the ability to penetrate deep muscle layers. In all samples, cyanides diffused to a depth of at least 1 cm. In the second layer (1–2 cm), they were absent in one test, and in the third layer (2–3 cm), traces of cyanides were not found in 4 out of 16 tests. Autolysis and putrefaction can be excluded as important sources of endogenous HCN in the 24 h experiments. The specimens did not show putrefaction changes, and the concentration of HCN in each trial gradually decreased from the superficial layer. Furthermore, the thermal coagulation of the superficial muscle layer did not suppress the HCN diffusion. Our findings demonstrate that the thermal coagulation of the superficial muscle layer favored cyanide penetration into deeper layers and blood located below the 4.5 cm muscle layer. The diffusion of cyanides is likely to be facilitated by the dehydration of proteins in the coagulated muscle and the shift of water deeper into the muscle. However, a thicker layer of adipose tissue greatly limited HCN diffusion into the blood and muscles. This conclusion can be drawn from the comparison of the experiments performed according to pattern number 1 and pattern number 2 (for CO and HCN). 

## 6. Limitations of the Study

Since it was impossible to consider the effects of high temperature on the acceleration of diffusion (in fire conditions), a very long exposure time was accepted (i.e., 24 h). In real fire cases, the diffusion of toxic gases into the body is undoubtedly faster and the time of exposure is substantially shorter as compared to the conditions of our experiment. The evaluation of these relations requires further studies with an experimental chamber of different designs. A slight diffusion in the case of extremely high CO and HCN concentrations under the conditions of a 24 h experiment indicate the negligibly low influence of the postmortem diffusion of those cases (especially into the deeper layers of the corpse) on the concentrations of COHB and HCN in real forensic settings. CO levels as high as 3000 ppm (0.3% vol.) were observed for some fires [38], and in some experimental fire settings, volume concentrations greater than 10% were observed [39], whereas HCN concentrations only reached 42 ppm [40]. However, this information should be considered very carefully, especially when investigating the tissues collected from heavily charred corpses.

## 7. Conclusions

In the cases of corpses brought out of fire conditions, the collection of muscle or blood from a depth of several centimeters should eliminate the risk of postmortem diffusion, all the while enabling the confirmation of intravital CO (even in charred corpses) along with HCN inhalation. The blood should not be collected from the vessels lying directly under the skin, especially in slim individuals without substantial subcutaneous fatty tissue layers. However, HCN may even diffuse into the profound muscles and vessels in the case of charred corpses with denudation of thermally coagulated muscles. In such cases, it may be a cause of false-positive diagnoses of fatal poisonings with this gas.

## Figures and Tables

**Figure 3:**
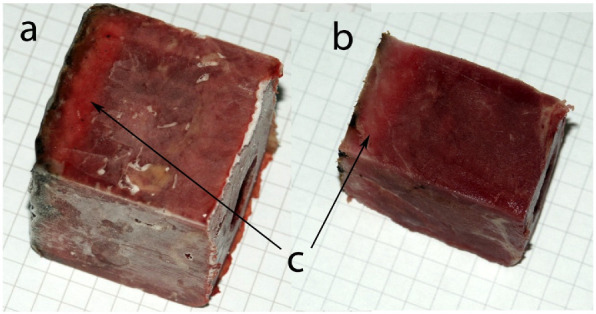
A frozen muscle fragment after the experiment with CO; pattern 4 with the superficially charred surface. (**a**) After removal from the chamber; (**b**) after cutting off 5 mm peripheral layers and superficial charring; (**c**) brighter, vividly red diffusion area.

**Table 1:** Concentrations (% sat.) of COHb in blood and COMb in 3 layers of muscles after the 24 h CO exposure.

Patterns of Tissue Layers	Skin	Fat	Blood	Muscle 0–1 cm	Muscle 1–2 cm	Muscle 2–3 cm	Blood
1	layer present	layer present	0.4	0.1	0.1	<0.1	0.3
			0.2	0.2	0.1	<0.1	0.2
			<0.1	0.4	<0.1	0.0	0.0
			<0.1	0.4	0.2	0.0	<0.1
2	layer present	layer absent	24.9	2.8	0.3	0.2	1.6
			28.2	2.2	0.2	0.2	0.8
			7.3	1.0	<0.1	0.0	<0.1
			16.2	1.9	0.1	0.0	1.1
3	layer absent	layer absent	layer absent	3.7	<0.1	0.0	0.3
				4.6	0.0	0.0	0.3
				6.5	0.1	0.1	1.6
				7.6	0.1	0.1	0.5
4	layer absent	layer absent	layer absent	8.2 *	<0.1	<0.1	<0.1
				5.8 *	<0.1	<0.1	0.3
				1.5 *	0.4	0.1	2.4
				1.6 *	0.3	0.1	1.0

*—superficially charred muscle (see Section 2.1).

**Table 2:** Concentrations of HCN in blood (mg/L) and 3 muscle layers (mg/kg) after the 24 h exposure at +30 °C.

Patterns of Tissue Layers	Skin	Fat	Blood	Muscle 0–1 cm	Muscle 1–2 cm	Muscle 2–3 cm	Blood
1	layer present	layer present	2.80	0.60	0.00	0.00	0.00
			2.43	0.08	0.02	<0.02	0.00
			0.74	0.06	0.04	<0.02	0.03
			0.99	0.05	0.02	<0.02	0.06
2	layer present	layer absent	20.50	2.61	1.23	0.00	0.00
			47.85	5.37	1.40	0.00	0.00
			48.91	1.16	0.30	0.06	0.00
			38.61	2.72	0.84	0.00	0.00
3	layer absent	layer absent	layer absent	17.26	7.19	0.08	0.02
				15.97	8.31	0.06	0.00
				15.32	13.63	2.86	0.30
				19.78	9.11	1.90	0.11
4	layer absent	layer absent	layer absent	13.92 *	11.00	2.60	1.99
				15.44 *	8.37	2.27	0.56
				16.79 *	4.80	0.81	0.36
				13.78 *	7.91	1.89	0.45

*—superficially charred muscle (see Section 2.1).

## Data Availability

Not applicable.

## Supplementary Materials

The following supporting information can be downloaded at: https://www.mdpi.com/article/10.3390/toxics10110707/s1, Video S1: Experiment-video.
